# Post-residency medical education fellowships: a scoping review

**DOI:** 10.1080/10872981.2021.1920084

**Published:** 2021-05-10

**Authors:** Mariel L. Cataldi, Margot Kelly-Hedrick, Julie Nanavati, Margaret S. Chisolm, Walsh Anne L.

**Affiliations:** a Public Psychiatry Fellow, Boston Medical Center, Boston, Massachusetts, USA; b Duke University School of Medicine, Durham, North Carolina, USA; c Clinical Informationist, The Welch Medical Library, Johns Hopkins School of Medicine, Baltimore, Maryland, USA; d Professor of Psychiatry and Behavioral Sciences, and of Medicine, Johns Hopkins University School of Medicine, Baltimore, Maryland, USA; e Assistant professor,Department of Psychiatry and Behavioral Sciences, Johns Hopkins University School of Medicine, Baltimore, Maryland, USA

**Keywords:** Medical education, fellowships training, clinician educator, post-residency fellowship, career development

## Abstract

The authors conducted a scoping review to investigate the structure, content, and potential impact of post-residency medical education fellowships. The authors searched eight databases to identify English-language articles describing longitudinal, post-residency medical fellowships that both focused on medical education and described the structure and content of the curriculum. The authors summarized the findings of each article and, for those articles that included a program evaluation, assessed the potential impact of the program via the Kirkpatrick’s Four-Level Training Evaluation Model and the Medical Education Research Study Quality Instrument. Nine articles, describing a total of ten post-residency medical education fellowships, met inclusion criteria. Half of the programs were dedicated medical education fellowships and half were medical education tracks within a subspecialty fellowship. The content and educational strategies varied, with no two programs having the same curriculum. Most programs most focused on teaching skills, adult learning theory, curricular development, and medical education research/scholarship. Most programs used project-based learning, workshops, and faculty mentorship as educational strategies. Six of the articles included an evaluation of their program(s), all of which suggested positive changes, at least at the level of fellow behavior (Kirkpatrick level 3), and designs limited the strength of any conclusions drawn. This scoping review highlights the variation among medical education fellowships and the need for common curricular components, as well as program evaluation, across and within these fellowships. Additional assessment at higher levels of trainee outcomes will help guide the creation and revision of medical education fellowships, and inform the development of a core curriculum shared across programs. Such a core curriculum could then serve as the foundation for a certification program, by which a medical educator’s expertise could be recognized, thus elevating medical education to the stature it deserves within the academic mission.

Clinician educators are active in a variety of roles, including teaching, curriculum design and assessment, education scholarship, innovation, and leadership [1,2]. Most receive no formal training to prepare them for these roles and are required to learn education-specific skills while managing clinical responsibilities. While more than half of all medical schools have some type of faculty development program focused on developing skills relevant to clinical educators, these programs are generally limited to those with faculty appointments [1,3].

Medical schools first created medical education fellowships in the late 1970s with the goals of improving faculty members’ teaching skills, advancing academic research, and transforming physicians into clinician educators [4]. These fellowships follow the model of other post-residency medical specialty training fellowships, focusing on the skills needed to have a successful career as a clinician educator, at the start of their career. These titles have also been used to describe faculty development programs [3,5]. For the purposes of this paper, we defined medical education fellowships as longitudinal, post-residency training programs whose primary focus is to prepare clinicians to be educators, following the training model of other specialty fellowships.

Medical education fellowships provide fellows protected time – before faculty appointment – to acquire the foundational knowledge and skills in medical education necessary to excel professionally. At the 2012 Academic Emergency Medicine Consensus Conference on Education Research, education leaders met to determine optimal training for education scholars and after an in-depth needs assessment, proposed a post-residency medical education fellowship prior to faculty appointment [6]. Proposed benefits to training prior to faculty appointment, as opposed to after faculty appointment, include starting one’s career with mastery of skills needed for a successful academic career, an existing research focus and academic productivity, an established mentor relationship [6].

The number of medical education fellowship programs increased significantly since they were first implemented, as the number of graduating medical students and the demand for skilled teachers to train the future generation of doctors also grew [3]. The benefits of these programs for both individuals and institutions are multiple. Individual benefits include enhanced desire for a career in academic medicine; increased confidence and self-efficacy as educators; improved skills in teaching, curriculum development, and assessment; education scholarship; and ongoing professional development [3,4,7–9]. Demonstrated institutional benefits include creation of a cadre of skilled teachers and educational leaders, increased educational projects/activities, and improvement in the educational community [5,8,10].

Current literature supports the importance and benefits of medical education fellowships [3–10]. However, no standardized criteria exist for these fellowships [10]. Post-residency medical education fellowships are diverse and heterogeneous [10]. These fellowships differ in many ways, including which medical specialties they serve and program duration. Perhaps most importantly, these programs differ in curricular content. To our knowledge, no review that summarizes post-residency medical education fellowship programs and their curricular content exists. We conducted a scoping review to investigate the structure and content, as well as any potential impact, of post-residency medical education fellowships described in the literature, to help guide the development of future programs.

## Methods

**Figure 1.**
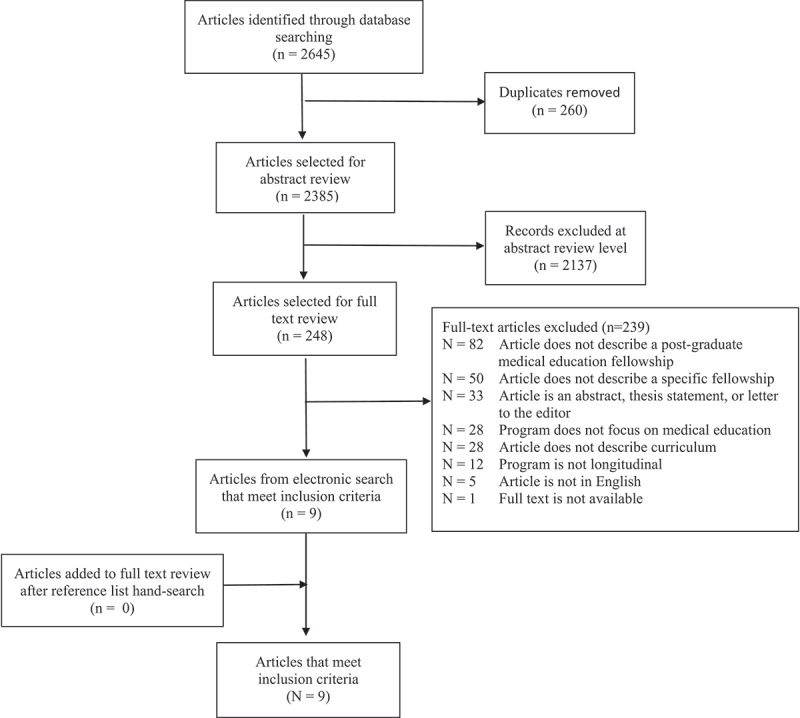
Flowchart of search strategy

Following the Joanna Briggs Institute guidelines [11] for conducting a scoping review, we pre-searched Medline (PubMed) and PsycINFO (EbscoHost) databases and identified relevant citations. We used these citations to create a final search strategy, which was developed by the study’s medical informationist (JN) in collaboration with the rest of the team. We ran the final search on 5 June 2019, in the following databases: Medline (PubMed), Embase (Embase.com), The Cochrane Library (Cochrane Database of Systematic Reviews, Cochrane Central Register of Controlled Trials (CENTRAL), Cochrane Methodology Register), ERIC (EbscoHost), CINAHL (EbscoHost), PsycINFO (EbscoHost), and Web of Science (Science and Social Science Citation Index). For the search strategies designed for Medline (PubMed), the Cochrane Library, PsycINFO, ERIC, CINAHL, and Embase, we identified controlled vocabulary terms for each concept and combined these with keyword synonyms. We searched Web of Science using keyword terms only. Results were limited for academic journals in PsycINFO, ERIC, and CINAHL (see Figure 1 for detailed search strategy). We also conducted a hand search of references of all included papers, through which we identified no additional articles.

We transferred titles and/or abstracts of identified articles to Covidence [12] for further review. Two team members independently reviewed all article abstracts to determine whether they were relevant to the research question. A third team member resolved discrepancies. Next, two team members independently reviewed the full-text versions of the relevant articles, with an additional team member again resolving discrepancies. We excluded articles based on the following criteria: article is not in English, article is an abstract, thesis statement, or a letter to the editor, article does not describe a fellowship or faculty development program, program does not focus on medical education, program does not occur after completion of residency training, program is not longitudinal (defined as the same cohort participating in a program of at least 3 months duration), or the article does not describe core contents or structure of the program’s curriculum. In addition to fellowships focused on medical education, we chose to include longitudinal medical education tracks within primarily clinical or research fellowship programs (of at least one year’s duration) that also met the other inclusion criteria for medical education fellowships. Although we initially included fellowships and faculty development programs, we found that the majority of those initially included were faculty development programs and later refined our inclusion criteria to include only post-residency fellowships in order to address the lack of literature specifically focused on these programs. Two study team members independently extracted relevant data from the final set of included articles.

Two team members assigned Kirkpatrick’s Four Levels of Training Evaluation to each article based on the outcomes that were reported The Kirkpatrick model assesses programmatic effects or effectiveness, with 4 levels of outcomes including participant’s reaction (Level 1), participant’s learning (Level 2), change in behavior (Level 3), and program results (Level 4) [13]. The quality of each quantitative study was assessed using the Medical Education Research Quality Instrument for Quantitative Studies (MERSQI), calculating the percentage of possible points and adjusting to a standard denominator of 18, with higher scores indicating higher quality of study design [14]. Programs were first evaluated independently by two coders, then discrepancies were discussed and resolved together.

## Results

### Description of search results

Our initial database search identified 2645 article from the databases. After removal of duplicates, a total of 2385 articles remained. We excluded 2137 articles after screening of title and abstracts, leaving 248 articles for full-text review. After full-text review, we determined that nine articles [15–23], for a total of ten post-residency medical education fellowships, met our inclusion criteria. We identified zero additional articles for full-text review after a hand-search of references (Figure 1).

### Fellowship structure and funding

The structure and funding of the fellowships is summarized in Table 1. Half of the fellowships described were dedicated fellowship programs for medical education [16,18,19,23] and half were medical education tracks within a subspecialty fellowship [15,17,20–22]. All fellowships included trainees from only one single specialty except for one fellowship that included trainees from two different specialties [16]. Specialties included cardiology, dermatology, emergency medicine, general internal medicine, geriatrics, pediatrics, pulmonary and critical care medicine, and joint medicine and anesthesiology. Most of these fellowships [15,16,18–23] were small, only associated with one university, and typically limited to 1–2 fellows per year, although one program was affiliated with four separate institutions and trained up to 10 fellows per year [17]. The duration of most dedicated medical education fellowships was 1–2 years. In three fellowships, trainees also completed either a master’s degree in education or a medical education certificate program [19,23]. Four of the fellowships were funded by grants [17,19,21,22], one by institutional funding [15], and one by philanthropy [18]. Funding was not described for four fellowships [16,20,23].

**Table 1.** Post-residency medical education fellowship demographics

Program Characteristics	Category	N (%)
	Total	10 (100)
Location	USA	9 (90)
	UK	1 (10)
Affiliation	One university/institution	9 (90)
	Multiple institutions	1 (10)
Program Type	Dedicated education fellowship	5 (50)
	Track of existing fellowship	5 (50)
Specialty	Cardiology	1 (10)
	Dermatology	1 (10)
	Emergency Medicine	2 (20)
	Family Medicine	1 (10)
	General Internal Medicine	1 (10)
	Geriatrics	1 (10)
	Pediatrics	1 (10)
	Pulmonary & Critical Care Medicine	1 (10)
	Joint Medicine and Anesthesiology	1 (10)
Number of Fellows	1	2 (20)
	2	1 (10)
	>10	1 (10)
	Not stated	6 (60)
Duration	1 year	4 (40)
	2 years	3 (30)
	Not stated	3 (30)

### Curricular content and educational strategies

The specific curricular content areas and educational strategies varied for each fellowship, though there were some similarities between fellowships. We summarized the curricular content and education strategies of each fellowship in Table 2. Curricular content common in most programs included teaching skills, curriculum development, adult learning theory, medical education research/scholarship, and career development. A minority of the programs included leadership as an area of focus [15,18,22]. No single content area was included in all fellowships. Project-based learning was the most commonly used pedagogical method, which was found in all programs except one [16]. Other methods included workshops and faculty mentorship. All fellowships used at least two co-occurring methods, and no single method was shared by all fellowships.

**Table 2.** Summary of post-residency medical education fellowships (N = 10)

Program Name	Curricular Content					Edsucational Strategies			Evaluation					
	**Teaching Skills**	**Adult Learning Theory**	**Curriculum Develop-ment**	**Medical education research/scholarship**	**Career Develop** **-ment**	**Workshops**	**Mentorships**	**Projects**	**Evaluated**	**Study Design**	**Outcome Measure**	**Results**	**Kirkpatrick Level**	**MERSQI**
University of Washington Pulmonary and Critical Care Fellowship Clinician Educator Track [15]	X	X	X	X	X	X	X	X	X	Not stated	Quantitative	Fellows assumed clinician educator roles Number of clinician educator faculty in the division has tripled	Level 4	
Whipps Cross University Hospital Medical Education Fellowship [16]	X					X								
The Donald W. Reynolds Consortium for Faculty Development to Advance Geriatrics Education (FD~AGE) Clinician-Educator Fellowship [17]	X	X	X	X	X		X	X	X	Reviewed annual progress reports	Mixed	Fellows assumed clinician educator roles	Level 3	13.2
Dermatology Fellowship for Academic Clinician Teachers at University of Pennsylvania [18]	X	X			X	X		X						
General Pediatrics Master Educator Fellowship (Cincinnati Children’s Hospital) [19]	X	X	X	X	X	X	X	X						
**Program Name**	**Curricular Content**					**Educational Strategies**			**Evaluation**					
	**Teaching Skills**	**Adult Learning Theory**	**Curriculum Develop-ment**	**Medical education research/scholarship**	**Career Develop** **-ment**	**Workshops**	**Mentorships**	**Projects**	**Evaluated**	**Study Design**	**Outcome Measure**	**Results**	**Kirkpatrick Level**	**MERSQI**
Medical Educator Pathway in Cardiology Fellowship at University of Michigan [20]	X	X	X	X		X		X						
Harbor-UCLA Family Medicine Medical Education Track [21]			X	X	X	X		X	X	Reviewed quality of written documentation of eight projects Reviewed number of new curricular opportunities	Mixed	All projects fulfilled between six and eight of the criteria set by expert reviewers Fellows implemented projects which created new or improved curricular opportunities	Level 3	10
Johns Hopkins GIM Fellowship Medical Education Track [22]	X	X	X	X		X	X	X	X	Reviewed CVs	Quantitative	Increase in total publications and publications with fellows as first authors Fellows assumed clinician educator roles	Level 4	
Harbor-UCLA EM Medical Education Fellowship [23]	X	X	X	X	X		X	X	X	Not stated	Qualitative	Fellows assumed clinician educator roles	Level 3	
OHSU EM Medical Education Fellowship [23]	X	X	X	X	X		X	X	X	Not stated	Qualitative	Fellows assumed clinician educator roles	Level 3	

### Program evaluation

Six of the articles about these fellowships reported on the assessment of learner outcomes, as shown in Table 2[15,17,21–23]. Among these, the design and outcome measures of each study were heterogeneous and often not clearly described. Two of the programs had qualitative outcome measures, two had mixed outcome measures, and two had quantitative outcome measures. Fellow satisfaction, post-fellowship clinician educator jobs, and scholarship productivity were evaluated by four programs [19,21–23]. Fellows gave positive feedback about their experience in the programs and felt the programs’ content was relevant and beneficial to their careers [17,22,23]. Additionally, scholarship productivity improved and most fellows obtained clinician educator jobs after fellowship.

Four studies reported outcomes that we categorized as Kirkpatrick level 3, indicating a change at the level of participant behavior, with fellows assuming clinician educator roles after fellowship as the most common [17,21,23]. Two studies reported institutional outcomes, categorized as Kirkpatrick level 4 [15,22]. These included an increase in the number of clinician education faculty in the department [15], and an increase in the total publications [22], as well as increase in the publications with fellows as first authors [22]. While four studies had quantitative outcomes, only three described the evaluation [17,21,22], for which we were able to calculate MERSQI scores. The average MERSQI score was 11.7. The most common reduction in score was due to study design and sampling, as most studies evaluated one group, at a single site, without a pretest or control group.

## Discussion

The goal of this review was to investigate the structure and content of post-residency medical education fellowships described in the literature. We found that medical education fellowships vary in terms of structure, curricula, instructional methods, and evaluation. Most are small, based at one institution, and offered through one department. This diversity of structure may be due to the diverse values and needs of the institution and/or department in which they reside.

While we found no standard curricular content among medical education fellowships, a few commonalities did emerge, and we recommend the following best practices based on the findings of this review when creating a post-residency medical education fellowship. Institutions should consider their own needs and resources when creating the structure of the fellowship. For instance, if a robust department-based fellowship exists and a need for additional education training is identified, the department may choose to add a medical education track to the existing fellowship rather than create an independent medical education fellowship. Funding opportunities may also determine the structure of the fellowships. We recommend institutions draw on curricular commonalities of existing fellowships, including teaching skills, curriculum development, adult learning theory, medical education research/scholarship, and career development, as well as make scholarship and projects a focus, as this was the most commonly identified benefit. We advise programs to consider opportunities for evaluation, especially at higher levels such as change in behavior or patient outcomes, and outcomes that should be evaluated to continue to secure funding.

In the national survey by Thompson et al, (2011) which focused on faculty development programs, less than half of programs allow fellows to participate [3]. Most studies focused on post-residency medical education fellowships describe a single program. While previous studies have compared multiple post-residency medical education fellowships [10], our review is the first to describe the structure and content as well as the outcomes of these fellowships. We hope this compilation and comparison of existing post-residency medical education fellowship and medical education tracks within fellowships brings increased clarity to the field and will inform the creation, revision, and evaluation of medical education programs.

This review has inherent limitations, including the ability to only capture the subset of medical education fellowships that have reported on their programs’ structure and content. In addition, because most fellowships are small in number, newer fellowships may not have an adequate sample size for evaluation or sufficient data for publication. Another limitation, which may also be a strength, is that we only included medical education fellowships and medical education tracks of clinical specialty-based fellowships. This is a limitation in the sense that other related and relevant programs – such as faculty development programs – are not be identified; however, it is a strength that this review focuses on programs designed for participants who have just finished their residency training. Additionally, some articles included descriptions and evaluations of both medical education fellowships and faculty development programs at the same institution without distinguishing which content was included in which type of program.

Our review confirmed that medical education fellowships confer both institutional and individual benefits, as has been described previously in the literature [3,5,7–10]. However, almost half of the fellowships described in the literature included no evaluation component, and most evaluated had were limited by the quality of study designs. Medical education fellowships require a significant amount of resources. Opportunities exist to evaluate the impact of these programs in general, as well as to identify which specific components are most beneficial. The impact of medical education fellowships not only needs to be assessed, but the outcome measures should extend beyond participant satisfaction. Additional assessment of higher levels of outcomes can not only guide the creation and revision of medical education fellowships, but also inform the development of a core curriculum shared across programs. Such a core curriculum could then serve as the foundation for a certification program, by which a medical educator’s expertise could be recognized, thus elevating medical education to the stature it deserves in the academic mission.

## References

[1] Sherbino J, Frank J, Snell L. Defining the key roles and competencies of the clinician–educator of the 21st century. Acad Med. 2014;89(5):783–8. 24667507 10.1097/ACM.0000000000000217

[2] Steinert Y, Naismith L, Mann K. Faculty development initiatives designed to promote leadership in medical education. A BEME systematic review: BEME guide no. 19. Med Teach. 2012;34(6):483–503. 22578043 10.3109/0142159X.2012.680937

[3] Thompson BM, Searle NS, Gruppen LD, et al. PMC3071874; A national survey of medical education fellowships. Med Educ Online. 2011;16:16. . 10.3402/meo.v16i0.5642 PMC3071874 21475643

[4] Bland CJ, Hitchcock MA, Anderson WA, et al. Faculty development fellowship programs in family medicine. J Med Educ. 1987;62(8):632–641. 3612725 10.1097/00001888-198708000-00003

[5] Searle NS, Hatem CJ, Perkowski L, et al. Why invest in an educational fellowship program? Acad Med. 2006;81(11):936–940. 17065850 10.1097/01.ACM.0000242476.57510.ce

[6] Coates WC, Lin M, Clarke S, et al. Defining a core curriculum for education scholarship fellowships in emergency medicine. Acad Emerg Med. 2012 12;19(12):1411–1418. 23279248 10.1111/acem.12036

[7] Irby DM, Hodgson CS, Muller JH. Promoting research in medical education at the University of California, San Francisco, school of medicine. Acad Med. 2004;79(10):981–984. 15383359 10.1097/00001888-200410000-00019

[8] Lown BA, Newman LR, Hatem CJ. The personal and professional impact of a fellowship in medical education. Acad Med. 2009;84(8):1089–1097. 19638779 10.1097/ACM.0b013e3181ad1635

[9] Wilkerson L, Hodgson C. A fellowship in medical education to develop educational leaders. Acad Med. 1995;70(5):457–458. 10.1097/00001888-199505000-00064 7748433

[10] Coates WC, Runde DP, Yarris LM, et al. Creating a cadre of fellowship-trained medical educators: a qualitative study of faculty development program leaders’ perspectives and advice. Acad Med. 2016;91(12):1696–1704. 26826070 10.1097/ACM.0000000000001097

[11] Peters MDJ, Godfrey C, McInerney P, et al. Chapter 11: scoping Reviews (2020 version). In: Aromataris E, Munn Z, editors. JBI manual for evidence synthesis. JBI; 2020.

[12] Covidence <https://www.covidence.org/reviews/active>. cited 2019 115

[13] Yardley S, Dornan T. Kirkpatrick’s levels and education ‘evidence’. Med Educ. 2011;46(1):97–106. 10.1111/j.1365-2923.2011.04076.x 22150201

[14] Reed DA, Beckman TJ, Wright SM, et al. Predictive validity evidence for medical education research study quality instrument scores: quality of submissions to JGIM’s medical education special issue. J Gen Intern Med. 2008;23(7):903–907. 18612715 10.1007/s11606-008-0664-3 PMC2517948

[15] Adamson R, Goodman RB, Kritek P, et al. Training the teachers. the clinician-educator track of the University of Washington pulmonary and critical care medicine fellowship program. Ann Am Thorac Soc. 2015;12(4):480–485. 25763811 10.1513/AnnalsATS.201501-032OT

[16] Campbell S, Cifelli P. So you want to be a medical education fellow. Ulster Med J. 2018;87(2):127–128. 29867272 PMC5974646

[17] Heflin MT, Bragg EJ, Fernandez H, et al. The Donald W. Reynolds consortium for faculty development to advance geriatrics education (FD~AGE): a model for dissemination of subspecialty educational expertise. Acad Med. 2012;87(5):618–626. 22450185 10.1097/ACM.0b013e31824d5251

[18] James WD. On the importance of the clinician-educator. dermatology fellowship for academic clinician teachers. Arch Dermatol. 1998;134(2):151–153. 9487206 10.1001/archderm.134.2.151

[19] Klein M, O’Toole JK, McLinden D, et al. Training tomorrow’s medical education leaders: creating a general pediatric master educator fellowship. J Pediatr. 2013;162(3):440–441.e1. 23438912 10.1016/j.jpeds.2012.12.041

[20] Konerman MC, Alpert CM, Sinha SS. Learning to be a clinician-educator: a fellow-driven curricular reform. J Am Coll Cardiol. 2016;67(3):338–342. 26796400 10.1016/j.jacc.2015.11.032

[21] Snyder S. A program to teach curriculum development to junior faculty. Fam Med. 2001;33(5):382–387. 11355650

[22] Thomas PA, Wright SM, Kern DE. Educational research at Johns Hopkins University school of medicine: a grassroots development. Acad Med. 2004;79(10):975–980. 15383358 10.1097/00001888-200410000-00017

[23] Yarris LM, Coates WC. Creating educational leaders: experiences with two education fellowships in emergency medicine. Acad Emerg Med. 2012;19(12):1481–1485. 23240922 10.1111/acem.12042 PMC3537510

